# Microbial transformation of PEG 400 by *Pseudomonas stutzeri*: implications for environmental remediation

**DOI:** 10.3389/fmicb.2025.1692604

**Published:** 2025-11-27

**Authors:** Mijhail Reyes-Bocanegra, Ever Piundo-Ponce, Fabian Bello-Yupanqui, Walter Rojas-Villacorta

**Affiliations:** 1 Facultad de Ingeniería y Arquitectura, Escuela de Ingeniería Ambiental, Universidad Cesar Vallejo, Trujillo, Peru; 2 Facultad de Ingeniería y Arquitectura, Escuela de Ingeniería Mecánica Eléctrica, Universidad Cesar Vallejo, Trujillo, Peru; 3 Grupo de Biotecnología Microbiana y Vegetal, Dirección de Investigación, Universidad Cesar Vallejo, Trujillo, Peru

**Keywords:** polyethylene glycol 400, biodegradation, polymer, *Pseudomonas stutzeri*, microbial bioremediation

## Abstract

Polyethylene glycol 400 (PEG 400) is a synthetic polymer that has found wide application in various industries. Despite its low toxicity, its persistence and mobility in aquatic ecosystems pose significant environmental risks. This study evaluated the ability of *Pseudomonas stutzeri* grow in minimal saline medium (MSM) using PEG 400 as the sole source of carbon and energy. Structural modifications of the polymer were analyzed by FTIR spectroscopy to assess its potential application in environmental remediation. Regarding methodology, the growth and the formation of clear zones were first evaluated in a solid medium supplemented with PEG 400. Subsequently, liquid medium systems were established in which the bacteria were inoculated and physicochemical parameters (pH, redox potential, and dissolved oxygen) and growth kinetics were monitored for a period of 30 days. The alterations and modifications in the structure were evaluated by means of FTIR spectroscopy. The temperature (28 °C) was maintained at a constant level throughout the evaluation period. The results demonstrated sustained bacterial proliferation in both systems (2% and 5%) across both solid and liquid media. Clear zones were observed in the solid medium. The growth rate (μ = 0.35 days^−1^) exhibited a higher value in the system with 2% PEG 400, while the 5% system demonstrated more stable proliferation. The pH levels of the systems remained within the slightly alkaline range (7.48–7.90). Fourier transform infrared spectroscopy (FTIR) analysis revealed structural alterations in the polymer at the conclusion of the treatment process. These alterations included the rupture of ether bonds and the subsequent formation of carbonyl groups. The findings highlight the potential of *P. stutzeri* to degrade synthetic polymers under laboratory conditions, supporting its application in bioremediation of contaminated water. The study also contributes to SDG 13 on climate action.

## 1 Introduction

Plasticizers are additives employed to enhance the properties of plastics. However, they have become a subject of environmental concern due to their persistence and widespread use (Manatunga et al., 2024). Among plasticizers, polyethylene glycol is particularly noteworthy. Polyethylene glycol 400 (PEG 400) is a hydrophilic synthetic polyether characterized by repeating units of -CH_2_CH_2_O- and terminal hydroxyl groups (HO–[CH_2_CH_2_O]n–H), where n determines its molecular weight and, consequently, the physical state of the polymer. PEG 400 has a low molecular weight of approximately 400 Da and is widely used in the pharmaceutical, medical, and cosmetic industries due to its hygroscopic, non-volatile, and non-toxic properties (Geisler et al., 2025; Kawai, 2010; Kawai and Schink, 1987).

The polymer PEG has been commercially produced for the synthesis of detergents and polyurethanes (Kawai, 2005). These polymers exhibit low toxicity and do not cause skin irritation, characteristics that make them suitable for use in the manufacture of nonionic surfactants (Kawai, 2005). These surfactants are utilized in various industrial applications, including household detergents, agrochemicals, and food emulsifiers. The polymer is also used as an intermediate in the preparation of resins such as alkyd resin and polyurethane resin, as a component in the manufacture of lubricants, antifreeze, moisturizers, printing inks, adhesives, shoe polish, softeners, glues, and plasticizers. These products have the potential to contaminate ecosystems and accumulate in wastewater, which has led to environmental concerns and prompted research on their biodegradation (Kawai, 2005). It has been established that polyethylene glycol (PEG) molecules exhibit low sorption and high mobility within sandy soil environments, facilitating their movement through soil layers and access to groundwater (Castanho et al., 2009). This mobility can cause PEG 400 to reach various ecosystems, where its fate will depend on local conditions, such as the presence of other chemicals and microbial activity. With respect to its toxicity, PEG 400 itself is not directly harmful; however, its degradation products have the potential to be more hazardous (Nilsson et al., 2025). Its favorable physicochemical profile, which includes high water solubility, low volatility, and relative chemical inertness under environmental conditions, theoretically suggests low inherent toxicity. However, the environmental implications of prolonged and repeated discharges into water bodies require thorough evaluation (Fiume et al., 2020; Pietrelli et al., 2021).

Despite the low toxicity of PEG 400, there are some reports of sublethal alterations in aquatic organisms that could potentially be related to oxidative stress or subtle biochemical alterations following exposure to higher concentrations of PEG 400 (Gu et al., 2019; Nascimento et al., 2021). For instance, research on amphibian tadpoles has documented indications of oxidative stress and neurotoxicity at concentrations that, while exceeding typical environmental levels, could nevertheless be pertinent in the context of localized industrial spills or inadequately treated wastewater discharges (Nascimento et al., 2021; Pham Le Khanh et al., 2022).

The environmental fate of PEG 400 is determined by its chemical properties, primarily its high-water solubility and relatively low molecular weight, which facilitates its mobility in aquatic environments (Fiume et al., 2020; Traverso-Soto et al., 2016). Preliminary studies have demonstrated that wastewater bacteria possess the capacity to mineralize polyethylene glycol (PEG), while subsequent investigations have identified bacterial strains that are capable of biodegrading the polymer (Watson and Jones, 1977). The polymer PEG 400 demonstrates susceptibility to degradation under both aerobic and anaerobic conditions. In the initial phase of biodegradation, the process is influenced by physical and chemical factors, including temperature, pH level, and the presence of active microbial communities (Chen and Hu, 2015; Fiume et al., 2020). Biodegradation under anaerobic conditions has been demonstrated in experiments with acclimated anaerobic sludge, where a reduction in biochemical oxygen demand (BOD) and total organic carbon (TOC) has been observed, reaching a PEG removal rate of 90% after adequate hydraulic retention times (Chen and Hu, 2015). Despite these favorable biodegradation profiles, the degree of degradation is dependent upon the operating conditions in wastewater treatment facilities, suggesting that rigorous control and monitoring of the process are essential to ensure that industrial effluents containing PEG 400 are properly treated before discharge (Chen and Hu, 2015; Fiume et al., 2020).

Several studies have shown that certain types of bacteria found in soil, activated sludge, and industrial wastewater can break down a substance called PEG 400. These bacteria use PEG 400 as their only source of energy and carbon. Some types of *Pseudomonas* and *Flavobacterium* are especially notable (Chen and Hu, 2015). The *Pseudomonas putida* Egypt-15 strain has shown optimal growth in media with PEG-4000 and PEG 400 under conditions where oxygen is present (AbdEl-Mongy et al., 2018; Kawai and Schink, 1987). Preliminary studies have indicated that certain genera, including *Pseudomonas*, *Sphingomonas*, and *Flavobacterium*, have been observed to be consistently associated with the biodegradation of polyethylene glycol (PEG) in environmental samples (Haines and Alexander, 1975; Kawai and Schink, 1987; Kawai, 2002). The presence of these bacteria in many different environments and their ability to break down PEG shows how important they are for the environment and for treating wastewater (Hosoya et al., 1978; Kawai, 2012).

The degradation mechanisms involve oxidative processes involving enzymes that act on terminal hydroxyl groups. Aerobic bacteria have certain enzymes that help with the oxidation of primary alcohol groups to aldehydes. These enzymes, like membrane-bound dehydrogenase, are found in the periplasmic space. These intermediates are then oxidized by aldehyde dehydrogenase enzymes, which produce by-products with carboxyl groups. These by-products can break bonds with ether molecules. This process breaks down the polymer into smaller units, like ethylene glycol and diethylene glycol. These smaller units can then be broken down using a process called the glyoxylate pathway. Then, the by-products can be completely oxidized in the respiratory chain linked to the periplasmic space membrane (Kawai and Schink, 1987; Kawai, 1995; Kawai, 1997; Kawai, 2002; AbdEl-Mongy et al., 2018 The anaerobic degradation of polyethylene glycol (PEG) through fermentative or syntrophic processes has been the subject of extensive research. One notable example is the bacterium *Pelobacter venetianus*, which is strictly anaerobic, captures PEG intracellularly, and then cleaves it using hydrolytic enzymes. However, further studies are necessary to elucidate the metabolic pathways and enzymes implicated (Suhaimi et al., 2021).

While PEG 400 is not generally regarded as a highly toxic substance. However, its widespread industrial use leads to frequent disposal through wastewater streams. This raises concerns about its persistence in aquatic environments. Consequently, in localized scenarios where its concentration is high, continuous monitoring and improved treatment strategies are essential (Fiume et al., 2020; Traverso-Soto et al., 2016). Degradation byproducts such as acetaldehyde, monoethylene glycol (MEG), diethylene glycol (DEG), and various organic acid may be toxic (Suhaimi et al., 2021). These compounds increase the organic load in wastewater, and conventional treatments do not always eliminate it completely. Microbial transformation using metabolically versatile bacteria like *P. stutzeri* to transform PEG 400 under controlled conditions. This initiative is expected to lay the groundwork for future environmental remediation strategies. This approach is consistent with the goal of SDG 13, which is to take action against climate change.

The present study evaluated the microbial transformation of PEG 400 by *P. stutzeri* under controlled conditions, using this polymer as the sole source of carbon and energy in a minimal saline medium. The physicochemical parameters, bacterial growth kinetics and structural modifications of the polymer were monitored using FTIR spectroscopy in order to determine its potential for use in environmental remediation applications.

Furthermore, recent studies on the microbial modification of natural polysaccharides, such as those extracted from *Artemisia sphaerocephala* (Kakar et al., 2021), and on the biodegradation of microplastics in aquatic environments by bacteria and fungi (Ullah et al., 2024) offer complementary perspectives that allow the transformation of PEG 400 to be contextualized within a broader framework of polymer biodegradation and environmental remediation. These references serve to underscore the ecological pertinence of the present study and its alignment with sustainable methodologies for the remediation of persistent pollutants.

## 2 Materials and methods

### 2.1 Bacterial culture

The *P. stutzeri* bacteria were provided by the laboratory of the Institute and Research Centers of César Vallejo University (Trujillo, Peru). The bacterial samples were reactivated in nutrient agar at an incubation temperature of 28 °C for a duration of 24 h (Geisler et al., 2025). A pure culture was obtained in slanted nutrient agar medium. Identification was performed by comparing the sequence with those deposited in the NCBI database. The closest match was found to be *Pseudomonas stutzeri* (accession number MT027239.1), which has previously been reported in studies on the generation of bioelectricity in microbial fuel cells (Rojas-Villacorta et al., 2023).

### 2.2 Culture media and PEG 400

The culture media used were Nutrient Agar (Oxoid, Basingstoke, UK) to reactivate the strains. Bushnell Hass Broth (HiMEDIA, Maharashtra, India) was used as the base medium for the degradation tests. This medium is composed (g/L) of: Magnesium sulfate (0.20); anhydrous calcium chloride (0.02); potassium dihydrogen phosphate (1.00); dipotassium hydrogen phosphate (1.00), ammonium nitrate (1.00) and ferric chloride (0.05). The medium used for colony counting was Agar Plate Count (Tm Media, India). Finally, the polymer to be degraded was PEG 400 (Sigma-Aldrich, Darmstad, Germany). The systems were made up of liquid medium, while the solid media had the minimum saline medium plus agar-agar (15 g/1 L) (Watson and Jones, 1977).

### 2.3 Pre-evaluation: growth and formation of clear zones in solid medium with PEG 400

The clear zone method was performed according to the protocol by Nademo et al. (2023), with modifications to the reagent used to reveal the clear zones. These tests enable the determination of the bacteria’s tolerance to the two concentrations (2% and 5%) of PEG 400, as well as the qualitative measurement of the enzymatic activity on the polymer. Initially, a pure culture was seeded in solid medium with PEG 400 (2% and 5%) and incubated at 28 °C for 2 weeks to observe the colonies. Only Control was incubated for 24–48 h. Consequently, a suspension of *P. stutzeri* was meticulously prepared in MMS. Petri dishes were prepared with Bushnell Hass agar plus PEG 400 (2% and 5% concentrations) as the sole source of carbon and energy. The wells were created in the solid medium, and 50 μL of the bacterial suspension was added. The samples were then subjected to an incubation process at a temperature of 28 °C for a duration of 24 h. Lugol’s iodine was introduced into the medium to reveal the degradation halos.

### 2.4 Design and operationalization of degradation systems

#### 2.4.1 Bacterial inoculum

The strain was initially cultivated in six nutrient agar plates from a pure culture. From these culture plates, a suspension was made in 500 milliliters of MMS. The inoculum were obtained from the suspension by adjusting them to McFarland tube No. 1 (1 × 10^8^ cells/mL).

#### 2.4.2 Biodegradation systems

The procedure was based on that described by Geisler et al. (2025) with some modifications. The systems were prepared in 1L screw-top bottles. Two concentrations (2% and 5%) of PEG 400 were utilized in the study. Each concentration consisted of three bottles containing a mixture of MMS, PEG 400, and bacterial inoculum, along with a control group comprising a single bottle of MMS and PEG 400. A total of eight bottles were obtained for further analysis. Following the adjustment of the degradation systems, the samples were subjected to an incubation process at a temperature of 28 °C for a duration of 30 days.

#### 2.4.3 Measurement of physical-chemical parameters

The parameters were measured using a HI98194 multiparameter meter (Hanna, Smithfield, RI, United States). The parameters assessed included pH, oxidation-reduction potential (ORP) (mV), and dissolved oxygen (DO) (mg/L). Measurement were conducted at 5-day intervals for a period of 30 days (Geisler et al., 2025).

#### 2.4.4 Measurement of microbial growth using optical density (DO_600_) and growth kinetics

Degradation was evaluated indirectly by measuring the increase in optical density (OD) using a GENESYS™ 140/150 Vis/UV-Vis spectrophotometer (Thermo Scientific, Waltham, MA, United States). Optical density (OD) measurements are obtained at a wavelength of 600 nm (OD600) on a three-day cycle for a duration of 30 days. The absorbance data at 600 nm (OD600) was initially processed to select the first three measurements (up to day 6) as a reference. These measurements were then transformed into Ln(OD600). These were then graphed, and the slope was plotted to obtain the linear equation (y = a + xb), where x is the slope (μ), which corresponds to the growth rate (days^–1^) of *P. stutzeri* in PEG 400. The calculation of the doubling time (TD) was performed using the following formula:


$$t_{d}\,\left(\text{days}\right)=\frac{\mathrm{Ln}\,\left(2\right)}{\mu }$$

#### 2.4.5 FTIR analysis

The samples were analyzed by FTIR-ATR using the Nicolet™ iS50 FTIR Spectrometer (Thermo Scientific, Waltham, MA, United States). A 10-ml sample was extracted from each of the treated bottles. The samples were filtered (0.22 μm) to capture any microorganisms present and subsequently transferred to glass Petri dishes. The dishes were then subjected to a drying process at 45 °C in an incubator for a duration of 12 h, with the objective of removing residual water. Following the drying process, a semi-dry residue was observed. This residue was then meticulously scraped until a sufficient amount of sample was obtained for placement in the FTIR. The evaluation was conducted at the commencement and conclusion of the experiment.

### 2.5 Data analysis

The data were processed with three replicates for each measurement (*n* = 3), and the analyses were performed in IBM SPSS v27. Graphs were created in OriginPro 2024. Given the absence of normality in the data, as indicated by the Shapiro-Wilk test with a *p*-value less than 0.05, nonparametric tests were employed for data analysis. Friedman was applied for repeated measurements of pH, ORP, and OD. For OD600, the Kruskal-Wallis test was employed to compare the four groups (System 2%, System 5%, Control 2%, Control 5%), followed by Dunn’s test with Bonferroni correction. Furthermore, Mann–Whitney U was utilized to directly compare the 2% and 5% inoculated systems to evaluate the effect of concentration on bacterial growth. Mann–Whitney U was also used to compare ORP and OD between inoculated systems and controls. The significance level was set at α = 0.05.

## 3 Results

### 3.1 Growth in solid medium and formation of clear zones

**FIGURE 1:**
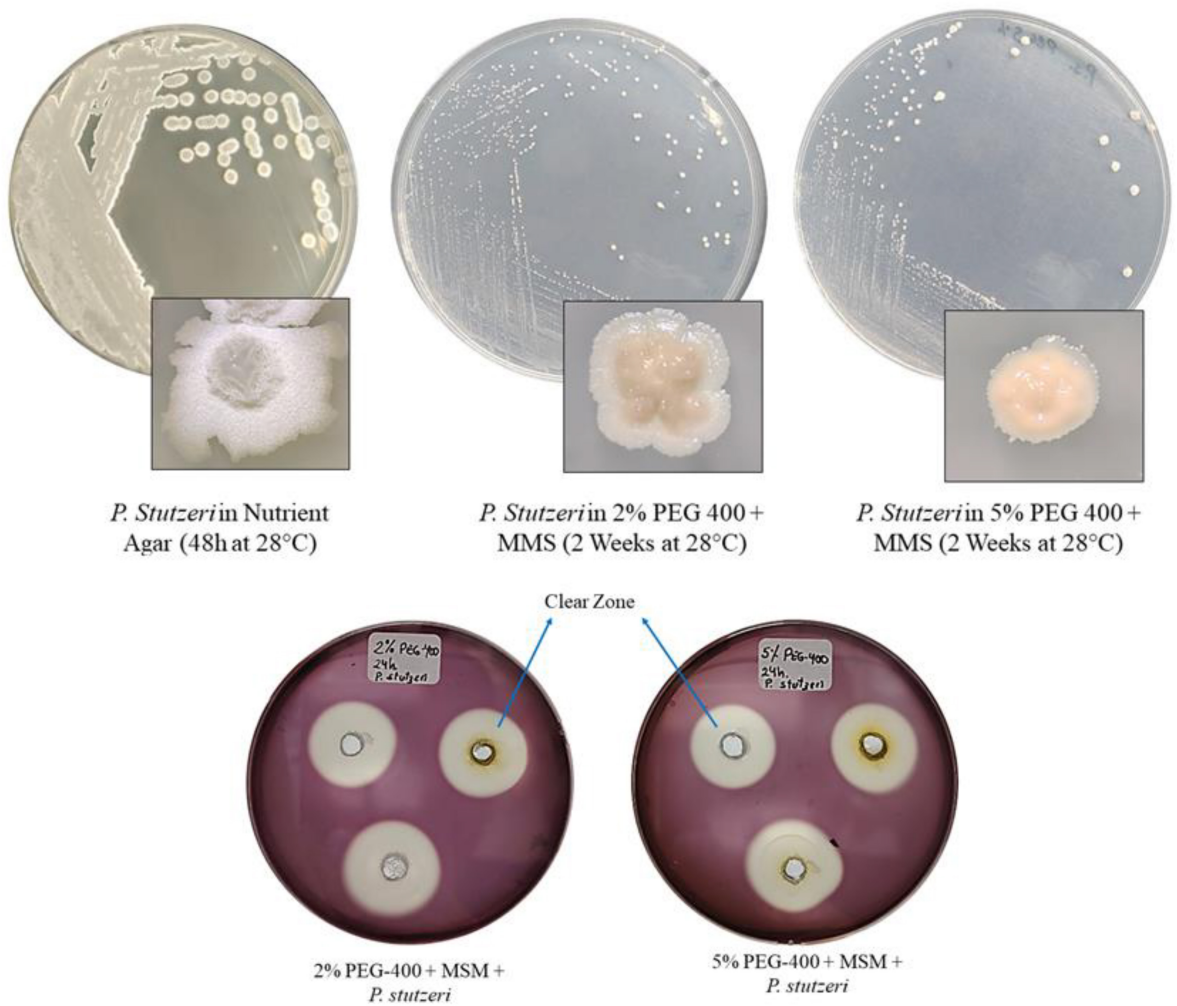
Growth of *P. stutzeri* in culture medium: Nutrient Agar; MMS + 2% PEG 400; MMS + 5% PEG 400. Clear zones in media with both concentrations of PEG 400 stained with Lugol’s iodine.

Figure 1 shows the growth of *P. stutzeri* in MMS medium plus PEG 400, at both 2% and 5%. As shown in Figure 1, the colonies appear small and round compared to those that grew in Nutrient Agar, where the colonies are crater-shaped after 24 h of incubation. Clear zones (or degradation zones) were observed in the solid media, with diameters of 23.7 and 23.3 mm for the 2% and 5% PEG 400, respectively.

### 3.2 pH variation

**FIGURE 2:**
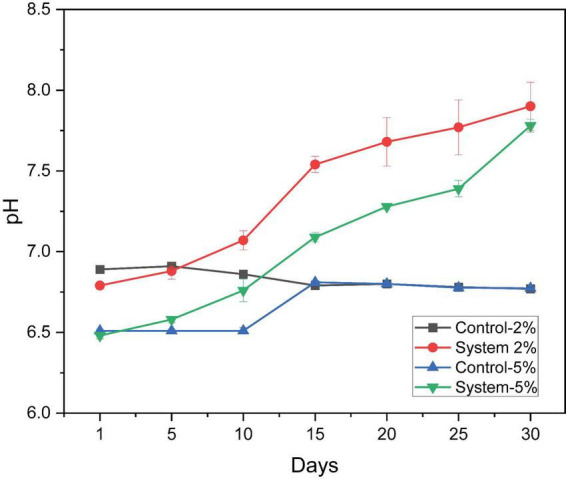
pH variation in degradation systems with *P. stutzeri* at two concentrations of PEG 400 (2 and 5%). Average ± SD (*n* = 3).

During the biodegradation process of PEG 400, the variation in pH was monitored as an indirect indicator of metabolic activity. Friedman’s nonparametric analysis revealed significant differences (X^2^ = 35.744, gl = 3, *p* < 0.001) in pH between the experimental systems (Control 2%, Control 5%, System 2%, and System 5%) at all time points evaluated (Figure 2). The mean ranges obtained indicate that the systems with *P. stutzeri* consistently presented higher pH values compared to the controls without inoculum. Specifically, the system with 2% PEG 400 exhibited the highest average range (3.71) in comparison to the 5% system, which demonstrated an average range of 2.64. The elevated value indicates a heightened metabolic activity associated with the process of biodegradation. In contrast, the controls with 2% and 5% PEG 400 exhibited lower average ranges of 2.19 and 1.45, respectively, indicative of stability in pH values and the absence of significant biological activity. In systems containing *P. stutzeri*, the pH level exhibited a progressive increase from values approaching neutrality to a slightly alkaline range (7.48–7.90) by the conclusion of the incubation period (Day 30). In contrast, the control groups-maintained stability at approximately 7 throughout the experimental course. The effect was more pronounced in the system with 5% PEG 400.

### 3.3 Redox potential (ORP) and dissolved oxygen (DO)

**FIGURE 3:**
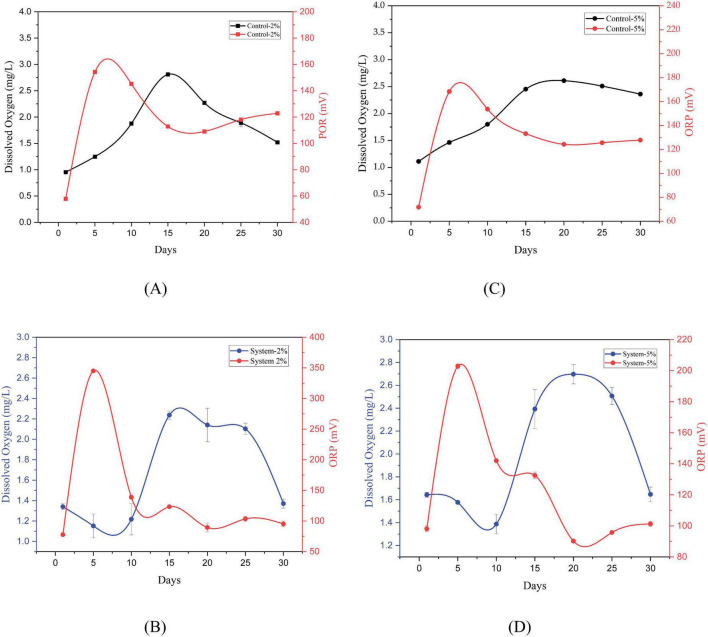
Evolution of dissolved oxygen and redox potential in control systems **(A,B)** and in P. stutzeri systems during the degradation of PEG 400 at 2% and 5% **(C,D).**

The evolution of redox potential and dissolved Oxygen was monitored for 30 days in systems with PEG 400 at two concentrations (2% and 5%), both in the presence of *P. stutzeri* and in controls without inoculation (Figure 3).

In systems with 2% PEG 400 (see Figures 3A, B), the redox potential of the controls increased from 57.83 ± 0.29 mV on day 1 to 154.23 ± 0.76 mV on day 5, with subsequent fluctuations and a final value of 122.80 ± 0.10 mV on day 30. The DO levels exhibited a marked increase from 0.953 ± 0.021 mg/L at the initial phase to a maximum of 2.810 ± 0.017 mg/L on day 15, subsequently declining to 1.520 ± 0.006 mg/L. In the systems inoculated with *P. stutzeri*, the ORP exhibited an initial reading of 77.67 ± 0.23 mV, which peaked on day 5 and concluded at 95.13 ± 4.62 mV. Concurrently, the DO levels experienced a decline, ranging from an initial value of 1.340 ± 0.030 mg/L to a minimum of 1.152 ± 0.117 mg/L on day 5. Thereafter, the DO levels exhibited an uptick, reaching a maximum of 2.237 ± 0.040 mg/L on day 15 and culminating at a final value of 1,370 ± 0.044 mg/L. Friedman’s analysis revealed substantial temporal variations for both DO (X^2^ = 77.345, df = 2, *p* < 0.001) and ORP (X^2^ = 65.278, df = 2, *p* < 0.001), with higher average ranges in the inoculated systems (DO = 3.71; ORP = 3.64) compared to the controls (DO = 2.19; ORP = 2.36).

In systems with 5% PEG 400 (see Figures 3C, D), the control exhibited an initial ORP of 71.83 ± 0.29 mV, which increased to 168.33 ± 0.91 mV on day 5 and decreased to 127.80 ± 0.10 mV on day 30. The DO levels initially registered at 1.110 ± 0.026 mg/L, subsequently reaching a maximum of 2.610 ± 0.014 mg/L on day 20, and concluding at 2.360 ± 0.014 mg/L. In the inoculated systems, the ORP exhibited an initial reading of 98.07 ± 1.93 mV, reaching a maximum of 202.83 ± 1.00 mV after 5 days. It subsequently decreased to 101.27 ± 1.59 mV by day 30. Concurrently, the DO level exhibited a fluctuation from 1.643 ± 0.021 mg/L on day 1 to a nadir of 1.387 ± 0.085 mg/L on day 10, followed by an ascension to a zenith of 2.697 ± 0.084 mg/L on day 20. Thereafter, it attained a state of stability at 1.647 ± 0.064 mg/L by the conclusion of the experiment (day 30). Friedman’s analysis revealed substantial temporal variations in both DO (X^2^ = 52.624, df = 2, *p* < 0.001) and ORP (X^2^ = 40.691, df = 2, *p* < 0.001), with higher mean values observed in the inoculated systems (DO = 2.64; ORP = 2.71) compared to their respective controls (DO = 1.45; ORP = 1.29).

Overall, the systems inoculated with *P. stutzeri* exhibited significant fluctuations in ORP and DO over the 30-day period (Friedman, *p* < 0.001), as evidenced by higher average ranges compared to the control groups. However, the Mann–Whitney U test revealed no statistically significant differences in either DO (2%: *U* = 198.0, *p* = 0.571; 5%: *U* = 201.5, *p* = 0.633) or ORP (2%: *U* = 186.0, *p* = 0.385; 5%: *U* = 183.0, *p* = 0.345).

### 3.4 Optical density (OD_600_) and growth kinetics

**FIGURE 4:**
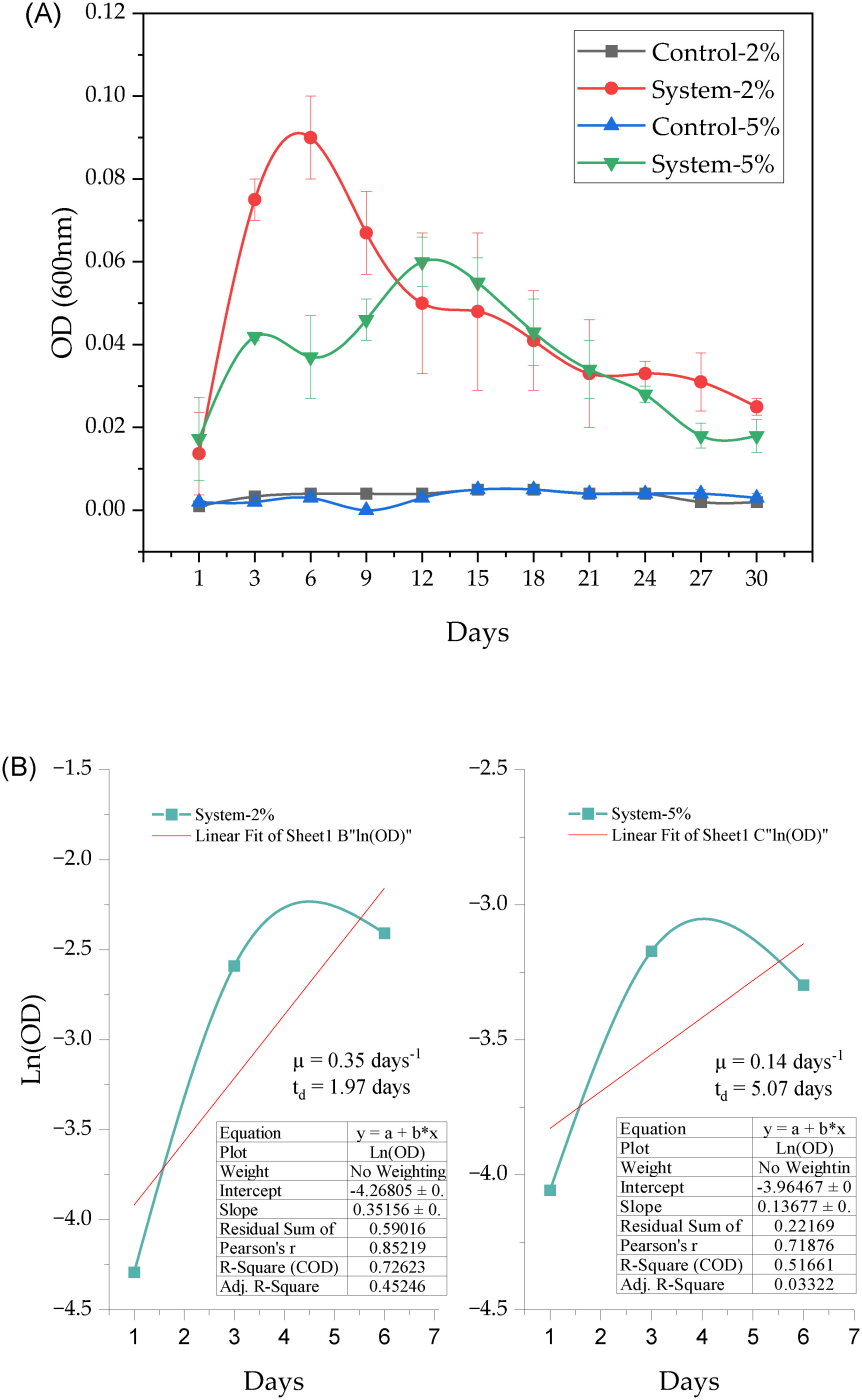
Growth of *P. stutzeri* in liquid medium supplemented with 2% and 5% PEG 400 under controlled conditions. **(A)** Growth dynamics based on optical density (OD600) over 30 days. **(B)** Linear fit of Ln(OD) to estimate growth rate (μ) and doubling time (td) in systems with 2% and 5% PEG 400.

Optical density at 600 nm (DO_600_) was evaluated in systems with PEG 400 (at 2% and 5%) inoculated with *P. stutzeri* and in control systems during the 30-day experiment (Figure 4). A subsequent Kruskal-Wallis statistical analysis for independent samples (*N* = 132) revealed significant differences between the four experimental groups (statistic = 99.842; df = 3; *p* < 0.001), with adjustments for ties. Pairwise comparisons were conducted using Dunn’s test with Bonferroni correction for multiple tests. This finding indicated substantial disparities between the inoculated systems (2% PEG 400 system and 5% PEG 400 system) and their respective negative controls (adjusted *p* = 0.000 in all cases). No statistically significant differences (adjusted *p* = 1.000) were identified between the two negative controls or between the inoculated systems. The OD_600_ values remained low and constant during the experimental period, while the inoculated systems showed progressive increases (Figure 4A).

The DO600 data were used to analyze the growth kinetics of the bacteria in the systems with 2% and 5% PEG 400, by linear adjustment of the Ln(DO_600_) data during the exponential phase (days 1 to 6). In the system with 2% PEG 400 (see Figure 4B), a specific growth rate (μ) of 0.35 days^–1^ and a doubling time (*t*_*d*_) of 1.97 days were obtained. The linear fit demonstrated a coefficient of determination (R^2^) of 0.726 and a Pearson correlation of 0.852. In the system with 5% PEG 400, the specific growth rate was 0.14 days^–1^, and the doubling time was 5.07 days. The linear fit demonstrated an R^2^ of 0.316 and a Pearson correlation of 0.516. These values indicate that the logarithmic model exhibited a superior fit to the data from the 2% PEG 400 system in comparison to the 5% system. A comparison of the two systems revealed that the 5% system exhibited a lower sum of residuals (0.22169) in comparison to the 2% system (0.59016).

### 3.5 FTIR analysis

**FIGURE 5:**
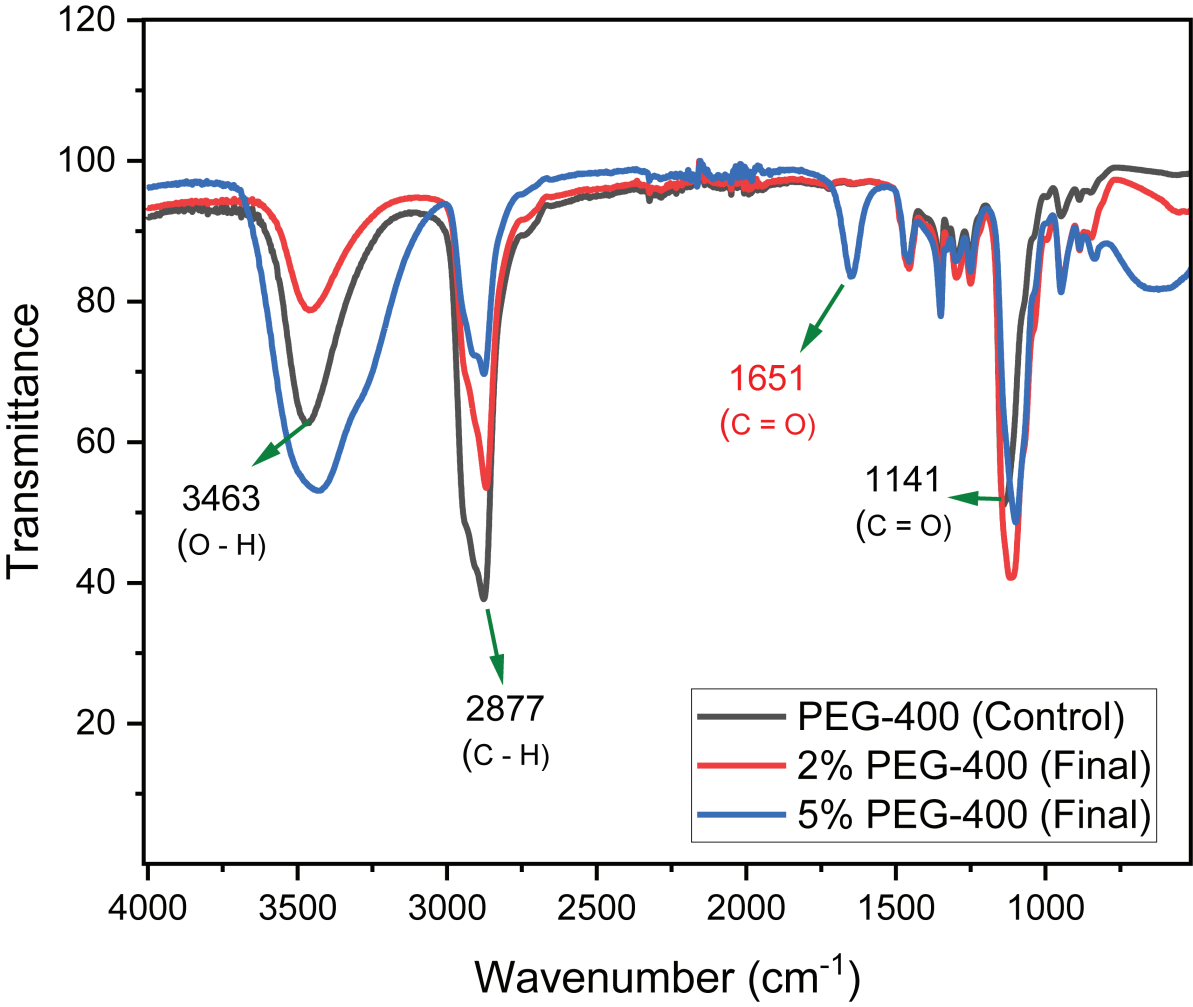
FTIR spectra of PEG 400 (2% and 5%) in control systems and systems inoculated with *P. stutzeri*.

The Figure 5, Fourier-trasform infrared (FTIR) spectroscopy revealed structural alterations in the transmittance bands of PEG 400 following a 30-day treatment with *P. stutzeri* at 28 °C. In the control sample (PEG 400 without inoculum), the spectrum exhibited the characteristic bands of the polymer, including a broad O-H stretching band within the range of 3500–3200 cm^–1^, an intense C-H stretching signal at 2880 - 2850 cm^–1^, and a C-O-C stretching band at 1100–1060 cm^–1^, situated within the fingerprint region (1500–500 cm^–1^). In the 2% and 5% PEG samples, a broadening and increase in intensity was observed in the O-H band (3500 - 3200 cm^–1^) and a decrease in the intensity of C-O-C (1100–1060 cm^–1^). Furthermore, the 5% sample exhibited a band at 1651 cm^–1^, positioned outside the fingerprint region. The C-H signal (2880–2850 cm^–1^) persisted in both treated concentrations, although with reduced intensity in the 5% sample.

## 4 Discussion

Figure 1 shows how *P. stutzeri* grows on nutrient agar and on MMS agar with 2% and 5% PEG 400 as the only carbon source. The growth of this bacterium in these media confirms that it does not require a lot of nutrients to survive. It can grow in conditions with limited nutrients and use carbon compounds that are unusual for other *Pseudomonas* species, such as ethylene glycol (Lalucat et al., 2006; Matías-Camacho et al., 2020). In all media, the colonies were whitish to creamy in color and lacked pigmentation; however, notable morphological differences were observed. On nutrient agar, the colonies were larger, crater-shaped or wrinkled, with thick, raised edges and a mucoid consistency. In contrast, in media containing PEG 400 (2 and 5%), there was a lower population density and punctate colonies, suggesting an effect related to the concentration of the polymer. This behavior can be attributed to both low nutrient availability and osmotic stress generated by PEG 400, which reduces water availability and causes altered growth patterns, including reduced colony size (He et al., 2006; Pérez-Padilla et al., 2025; Amini et al., 2017; Silva et al., 2007). The presence of clear zones in the supplemented media indicates that *P. stutzeri* possesses the necessary inducible enzymatic machinery to degrade PEG 400 at both concentrations (Suhaimi et al., 2021). These characteristics underscore the significance of the species in bioremediation processes, as its capacity to proliferate under restrictive nutritional and osmotic conditions confers an advantage for the degradation of polymeric contaminants (Amini et al., 2017).

The progressive increase in pH in systems with *P. stutzeri* suggests intense metabolic activity associated with the degradation of PEG 400 (Figure 2). This behavior stands in contrast to the stability observed in the control group, where the absence of significant variation (*p* < 0.001) reflects the lack of active biological processes. In systems 2% and 5%, there was a tendency toward slightly alkaline pH values (7.49–7.90), which suggests that these pH values may favor the degradation of PEG 400, as was found in the study by Jiang et al. (2013). In that study, the researchers used a strain of *Acinetobacter*, which achieved 83% degradation of the polymer in a pH range of 7.2–8.0. The capacity of *P. stutzeri* to degrade xenobiotic polymers in weak alkaline media has been corroborated by other studies employing different *Pseudomonas* strains in the degradation of phenanthrene and pyrene, with an optimal pH of 8.0 (Sharma et al., 2023; Yan-ling et al., 2010). A multitude of studies have indicated that neutral to slightly alkaline pH conditions (approximately 7.5) are conducive to the optimal efficiency of enzymatic activity. In particular, it has been reported that enzymes such as alcohol dehydrogenase, aldehyde dehydrogenase, and those capable of breaking ether bonds (all involved in PEG degradation) perform better in this pH range. Furthermore, the kinetics of polymer degradation tend to accelerate under these conditions (AbdEl-Mongy et al., 2018; Shuhaimi et al., 2021). In this context, the results obtained suggest that *P. stutzeri* not only induces favorable conditions for PEG biodegradation but is also a promising candidate for applications in environmental bioremediation processes.

In systems with 2% and 5% PEG 400 (see Figure 3), the ORP and DO dynamics demonstrated significant fluctuations during the 30-day evaluation period (Friedman analysis, *p* < 0.001), with higher average ranges observed in systems inoculated with *P. stutzeri* (DO = 3.71; ORP = 3.64) compared to controls (DO = 2.19; ORP = 2.36). However, the results of the overall comparisons did not reveal significant differences between the control group and the systems (2% and 5%), which may suggest that abiotic factors may be influencing the redox state of the medium. During the initial period, there was an increase in ORP and a decrease in OD in the systems. This phenomenon could be attributed to osmotic stress induced by PEG within the medium, which has been observed to generate reactive oxygen species (ROS). These ROS may contribute to the sustained elevated ORP values observed during the initial stages (Razavizadeh et al., 2019; Sahoo et al., 2020). Additionally, it has been established that ORP can be associated with DO; nevertheless, in the initial stages, there is a decline in this parameter. This observation may indicate that the bacteria are adapting to their substrate, which has not yet produced sufficient reducing by-products to elicit a decrease in ORP levels (Figures 3C, D). PEG 400 has been demonstrated to be susceptible to auto-oxidation through free radical reactions, which generate products that increase the oxidative state of the medium. This phenomenon has been observed to result in a high ORP value, particularly up to day 5 (Dokania and Joshi, 2015; Jannin et al., 2014). Furthermore, it has been established that under aerobic circumstances, these bacteria are capable of producing reactive oxygen species (ROS), which in turn impact the auto-oxidation of poly (ethylene glycol) (PEG 400) (Yang et al., 2011). In contrast, the biodegradation of PEG 400 by *P. stutzeri* involves the consumption of molecular oxygen, which leads to a decrease in DO in the medium (Gomes et al., 2023). The demand for oxygen consumption is associated with two processes: cellular respiration and extracellular enzyme production. Concurrently, as microbial activity advances, a decline in ORP is discernible, indicating a shift from an oxidative to a reductive milieu. Collectively, these findings reflect a multifaceted interaction between chemical and biological processes, wherein the potential auto-oxidation of PEG could generate a chemically oxidizing environment, while biodegradation by *P. stutzeri* leads to the depletion of dissolved oxygen necessary for respiration. This duality elucidates the discrepancy observed between DO and ORP in the early days and underscores the importance of incorporating abiotic controls to distinguish between redox phenomena of chemical and biological origin (Dokania and Joshi, 2015).

Figure 4A illustrates that *P. stutzeri* can proliferate in media augmented with polyethylene glycol (PEG) 400, in contrast to the control groups devoid of inoculum, in which no growth was observed. This observation validates the organism’s capacity to utilize the polymer as its exclusive carbon source, a trait that is analogous to that reported for *Acinetobacter* (Jiang et al., 2013), *Pseudomonas* sp., and *Sphingomonas* (Pan and Gu, 2007). This versatility is consistent with studies reporting its ability to degrade complex organic compounds (Kaczorek et al., 2012; Moscoso et al., 2015; Zhao et al., 2009), polymeric structures present in plastics (Wilkes and Aristilde, 2017), and polyethylene glycols of different molecular weights (200–13.500 Da) (Kawai, 2010; Kawai, 2012; Gates and Crook, 2024; Suhaimi et al., 2021). The capacity to metabolize PEG has been linked to various enzymes, including PEG dehydrogenase (PegA), aldehyde dehydrogenase (PegC), PEG carboxylate dehydrogenase (PCDH), and glutathione S-transferase (GST) (Ohta et al., 2005; Somyoonsap et al., 2008; Yamashita et al., 2004). In particular, Gates and Crook (2024) demonstrated the presence of polyethylene glycol (PEG) dehydrogenases in the *P. stutzeri* JA1001 strain. These enzymes oxidize the terminal hydroxyl groups of the polymer, generating compounds such as glyoxylic acid. These compounds are subsequently channeled into the citric acid cycle.

With regard to the conditions under consideration, the system with 2% PEG 400 exhibited accelerated and more substantial growth during the initial days, subsequently followed by a decline in viable biomass, likely attributable to nutrient depletion or the accumulation of toxic metabolites. Conversely, in the system with 5% PEG 400, growth exhibited greater moderation but was more sustained, which may be indicative of a more stable equilibrium between substrate consumption and cell viability. This pattern was confirmed by kinetic analysis (see Figure 4B), which revealed that the specific growth rate was higher in the 2% PEG group (μ = 0.3516 day^–1^) than in the 5% PEG group (μ = 0.1368 day^–1^). This suggests that lower concentrations favor faster initial proliferation, while higher concentrations promote slower but prolonged growth. The collective findings indicate that the response exhibited by *P. stutzeri* to PEG is predominantly influenced by its metabolic flexibility to adapt to the polymer, rather than by the absolute concentration of the polymer itself.

The FTIR spectrum of untreated PEG 400 exhibited the characteristic bands of the polymer (see Figure 5). These bands included C-H (–CH_2_–) stretching vibrations in the 2872–2923 cm^−1^ region, an intense C–O–C (ether) signal around 1089 cm^−1^, and multiple signals in the fingerprint region (1474–1287 cm^−1^) associated with –CH_2_ deformations. Collectively, these bands constituted the polymer’s characteristic spectral signature (Deygen and Kudryashova, 2016). After 30 days of incubation with *P. stutzeri* at 28 °C, notable structural changes were detected, such as the broadening of the band associated with O-H (–OH) stretching at 3500–3200 cm^−1^, suggesting the formation of new hydroxyl groups and greater interaction through hydrogen bonds, as commonly observed during polymer oxidation processes (Sandt et al., 2021). These two changes can be observed in both systems (PEG 400: 2 and 5%). Another change is the decrease in the intensity of the C–O–C band in the 1100–1060 cm^−1^ region. This is consistent with the partial breakage of ether bonds in the polymer chain. Similar spectral changes have been reported in PEG-treated systems and oxidized polymers (Han et al., 2022; Maulana et al., 2021; Sandt et al., 2021). In the system with 5% PEG 400, a new signal was detected at 1651 cm^−1^, which is attributed to the generation of carbonyl groups (C = O) as oxidation products. In previous studies, this band has been directly related to the generation of oxidized species such as PEG-disuccinate, which supports the idea that the polymer undergoes progressive chemical modifications during the biodegradation process (Chen et al., 2012; Gupta et al., 2015). However, given that this region corresponds to the Amida I protein band, a partial contribution from residual biomass cannot be excluded (Navea et al., 2005; Farag et al., 2015). These findings suggest that *P. stutzeri* plays a role in promoting oxidation processes, such as chain scission, within PEG 400. However, the presence of potential overlap in signals derived from cellular compounds underscores the necessity for complementary analytical methods to definitively determine the origin of the observed bands. Additionally, these processes can be correlated with OD_600_ and growth kinetics, suggesting a link between the metabolic activity of the bacteria and the chemical transformation of the polymer.

The study demonstrates the versatility and ability of *P. stutzeri* to grow in solid and liquid media using PEG 400 at 2% and 5% as the sole source of carbon and energy under controlled conditions. However, it is important to consider the limitations of the experimental design. The medium used for evaluation consisted exclusively of mineral salts, and the temperature was maintained at 28 °C, which does not accurately reflect natural conditions. A notable limitation of the study was the evaluation of only two concentrations (2% and 5%), which could be expanded to resemble values of possible environmental contamination scenarios. Conversely, while FTIR analysis revealed structural modifications, further research is necessary to detect and quantify intermediate or final metabolites, which may possess toxicological significance, as evidenced in select studies (Liu et al., 2017). The findings serve to reinforce existing knowledge about the metabolic versatility of the species and its potential application in the biodegradation of polymers that are widely used in industry. Similarly, the integration of physicochemical parameters (pH, ORP, and dissolved oxygen) with spectrophotometric analysis (FTIR) constitutes a methodological contribution that allows the microbial dynamics to be related to the chemical transformations of the polymer. Consequently, novel perspectives are emerging for the development of more efficient biotechnological strategies for the treatment of effluents contaminated by synthetic polymers.

These findings facilitate the establishment of a correlation between the transformation observed in PEG 400 and the broader processes of microbial modification of polymers. This approach is consistent with recent research on the biodegradation of microplastics in aquatic ecosystems, which has emphasized the role of cultured and uncultured bacteria in environmental mitigation strategies (Ullah et al., 2024). Similarly, advancements in the microbial transformation of natural biopolymers, such as the polysaccharides of *A. sphaerocephala* (Kakar et al., 2021), underscore the biotechnological potential of microorganisms in the modification of complex polymers. These references enable the contextualization of the present study within a broader framework of environmental remediation and applied biotechnology.

## 5 Conclusion

The results obtained in solid and liquid media suggest that *Pseudomonas stutzeri* can use PEG 400 as its sole source of carbon and energy, promoting structural transformations in the polymer, as observed in FTIR analyses. These findings support the metabolic versatility of the bacterium and highlight its potential for bioremediation applications of synthetic polymers widely used in industry. The physicochemical parameters, including pH, were found to be consistent with prior reports indicating that slightly alkaline conditions favor PEG degradation in minimal media. However, the growth kinetics did not demonstrate a significant dependence on the evaluated concentration. A synthesis of the research conducted under controlled conditions offers a robust foundation for elucidating the potential of this bacterium to contribute to the treatment of wastewater and other environments impacted by synthetic polymers. However, further research is necessary to achieve a comprehensive understanding of the subject. This research should include pilot-scale trials, metagenomic analyses to identify the genes and enzymes involved, and toxicological evaluations of the intermediate or final metabolites generated. Consequently, the findings of this study not only augment the existing body of knowledge concerning microbial polymer transformation processes but also align with the objectives of SDG 13, thereby offering novel perspectives for mitigating the environmental impact of emerging pollutants.

## Data Availability

The original contributions presented in this study are included in this article/Supplementary material, further inquiries can be directed to the corresponding author.

## Supplementary material

Supplementary Table 1 Detailed data corresponding to pH, ORP, dissolved oxygen (DO), and OD_600_ [Ln(OD600)] measurements in systems with PEG 400 (2% and 5%) inoculated with *Pseudomonas stutzeri* and their respective controls.
